# Cancer Impacts Prognosis on Mortality in Patients with Acute Heart Failure: Analysis of the EPICTER Study

**DOI:** 10.3390/jcm11030571

**Published:** 2022-01-24

**Authors:** Manuel Méndez-Bailón, Noel Lorenzo-Villalba, Miriam Romero-Correa, Esther Guisado-Espartero, Juan González-Soler, Jessica Rugeles-Niño, Angel Sebastián-Leza, Luis Ceresuela-Eito, Verónica Romaní-Costa, Angustias Quesada-Simón, Llanos Soler-Rangel, Almudena Herrero-Domingo, Luis Díez-García, José Alcalá-Pedrajas, María Villalonga-Comas, Emmanuel Andrès, Diego Gudiñ-Aguirre, Francesc Formiga, Oscar Aramburu-Bodas, Jose Arias-Jiménez, Prado Salamanca-Bautista, on behalf of EPICTER Study Group

**Affiliations:** 1 Hospital Clínico Universitario San Carlos, Instituto de Investigación Sanitaria (IdISSC), 28040 Madrid, Spain; manuelmenba@hotmail.com; 2 Service de Médecine Interne, Diabète et Maladies Métaboliques, Hôpitaux Universitaires de Strasbourg, 67000 Strasbourg, France; emmanuel.andres@chru-strasbourg.fr; 3 Hospital General de Riotinto, 21660 Huelva, Spain; mirimedd@hotmail.com; 4 Internal Medicine Department, Infanta Margarita Hospital, 14940 Cabra, Córdoba, Spain; guesm53@hotmail.com; 5 Complejo Hospitalario Universitario de Ourense, 32005 Ourense, Spain; jugosoler2@yahoo.com; 6 Hospital Universitario Central de Asturias, 33011 Oviedo, Spain; jessicarugeles@hotmail.com; 7 Hospital Universitario de Cruces-Barakaldo, 48903 Bizkaia, Spain; angel.sebastian@ehu.eus; 8 Hospital del Baix Llobregat, 08970 Barcelona, Spain; lmceresuela@gmail.com; 9 Hospital Mutua de Terrassa, 08221 Barcelona, Spain; vromani@mutuaterrassa.es; 10 Hospital Universitario La Paz, 28046 Madrid, Spain; angusquesada@telefonica.net; 11 Hospital Universitario Infanta Sofía, 28703 Madrid, Spain; llanossoler@gmail.com; 12 Hospital General Nuestra Señora del Prado, 45600 Toledo, Spain; almihd@hotmail.com; 13 Internal Medicine Department, Torrecárdenas Hospital, 04009 Almería, Spain; lfdiez@telefonica.net; 14 Internal Medicine Department, Pozoblanco Hospital, 14400 Pozoblanco, Córdoba, Spain; jnalcala58@hotmail.com; 15 Hospital Universitario Son Espases, 07120 Palma de Mallorca, Spain; mvillalongacomas@gmail.com; 16 Hospital Universitario Nuestra Señora de Candelaria, 38010 Tenerife, Spain; diegojgudino@hotmail.com; 17 Hospital Universitario de Bellvitge, 08907 Barcelona, Spain; fformiga@bellvitgehospital.cat; 18 Hospital Universitario Virgen Macarena, 41009 Sevilla, Spain; oscarab2000@gmail.com (O.A.-B.); jlariasvin61@hotmail.com (J.A.-J.); pradosalamanca@gmail.com (P.S.-B.); 19 Universidad de Sevilla, San Fernando, 4, 41004 Sevilla, Spain

**Keywords:** acute heart failure, cancer, mortality

## Abstract

Introduction: Heart failure (HF) and cancer are currently the leading causes of death worldwide, with an increasing incidence with age. Little is known about the treatment received and the prognosis of patients with acute HF and a prior cancer diagnosis. Objective: to determine the clinical characteristics, palliative treatment received, and prognostic impact of patients with acute HF and a history of solid tumor. Methods: The EPICTER study (“Epidemiological survey of advanced heart failure”) is a cross-sectional, multicenter project that consecutively collected patients admitted for acute HF in 74 Spanish hospitals. Patients were classified into two groups according to whether they met criteria for acute HF with and without solid cancer, and the groups were subsequently compared. A multivariable logistic regression analysis was conducted, using the forward stepwise method. A Kaplan–Meier survival analysis was performed to evaluate the impact of solid tumor on prognosis in patients with acute HF. Results: A total of 3127 patients were included, of which 394 patients (13%) had a prior diagnosis of some type of solid cancer. Patients with a history of cancer presented a greater frequency of weight loss at admission: 18% vs. 12% (*p* = 0.030). In the cancer group, functional impairment was noted more frequently: 43% vs. 35%, *p* = 0.039). Patients with a history of solid cancer more frequently presented with acute HF with preserved ejection fraction (65% vs. 58%, *p* = 0.048) than reduced or mildly reduced. In-hospital and 6-month follow-up mortality was 31% (110/357) in patients with solid cancer vs. 26% (637/2466), *p* = 0.046. Conclusion: Our investigation demonstrates that in-hospital mortality and mortality during 6-month follow-up in patients with acute HF were higher in those subjects with a history of concomitant solid tumor cancer diagnosis.

## 1. Introduction

Heart failure (HF) and cancer are currently the leading causes of death worldwide, with an increase incidence with age [1]. It has been proposed that cardiovascular disease (CVD) and cancer share multiple pathophysiological mechanisms, and their association is very common [2,3,4]. The cardiotoxic effects of antineoplastic agents lead to the development of the specialty of cardio-oncology to improve care of patients and avoid the development of HF that can appear years after treatment is completed. In addition, cancer and HF share multiple risk factors involved in their pathogenesis, such as diabetes mellitus and dyslipidemia [3,4].

In addition to the well-known cardiovascular risk factors, the hyperactivation of the renin–angiotensin–aldosterone system (RAAS), the sympathetic nervous system (SNS), and the natriuretic peptide system described in patients with HF is also an important pathophysiological mechanism involved in the development of cancer. SNS hyperactivity may lead to tumor development through the β-AR-dependent activation of stimulatory G protein–protein kinase A and β-arrestin-1 signaling, which promotes the accumulation of DNA damage and impeded repair [5]. Furthermore, the angiotensin receptor 1 (AT1R) is also expressed by different tumor cells [6]. 

Another contributing factor to HF and cancer is the presence of low-grade inflammation. Oxidative stress and inflammation are important aggravating factors in the development of HF. The decrease in nitric oxide bioavailability and reduction in protein kinase G activity in cardiomyocytes lead to diastolic dysfunction and thus HF, with preserved ejection fraction by decreasing nitric oxide bioavailability and, thereby, reducing protein kinase G activity in cardiomyocytes [4,7]. Left ventricular remodeling could result from this proinflammatory state. Regarding cancer, the persistent inflammatory state has been related to tumor cell proliferation and progression [4].

It is important to carry out further studies to evaluate the presence of both pathologies and to determine their natural evolution. In this respect, most studies have been carried out in the outpatient setting in patients with cancer to determine the incidence of HF or in patients with HF and the development of cancer during follow-up [8,9]. 

There are no studies that have evaluated the natural history and treatment received in the acute phases of HF decompensation in patients with a concomitant history of cancer. At the present time, little is known about the treatment received and the prognosis of patients with acute HF and cancer. In this sense, it is valuable to carry out this research to determine the clinical characteristics, palliative treatment received, and prognostic impact of patients with HF with a history of solid cancer with respect to patients with acute HF without cancer.

## 2. Material and Methods

### 2.1. Study Population

The EPICTER study (“Epidemiological survey of advanced heart failure”) is a cross-sectional, multicenter project that consecutively collected patients admitted for HF in 74 Spanish hospitals, including public and private hospitals, regardless of size. Patients were recruited in two periods (summer and winter). To avoid bias, hospitals began collecting data on the same day (1 June and 30 November 2016), during which all patients admitted to the Cardiology or Internal Medicine departments, Intensive Care Units, or any other service were included. Researchers at each center checked patients who met the inclusion criteria daily, and after the first day, each hospital continued to recruit patients on subsequent days until the required number was met. The minimum number of patients to be included for each hospital was pre-determined according to its bed size. Inclusion criteria were (1) age older than 18 years, (2) admission to the hospital room before 8:00 am on the day of data collection, (3) HF as the main cause of admission, including acute HF, acute pulmonary edema, acute coronary syndrome Killip III-IV, or cardiogenic shock. Exclusion criteria were (1) patients attended in the Emergency Department, but not yet admitted, and (2) patients who did not sign the informed consent. All patients received the usual treatments and medical care and were classified into two groups according to whether they met criteria for HF with and without solid cancer (sarcomas, lymphomas, and carcinomas). All data were sent to researchers at Virgen Macarena University Hospital, which acted as the coordinating center and where an internal audit was performed. 

### 2.2. Study Variables

Age, sex, comorbidities, and laboratory data were collected. The drugs administered and the procedures performed at admission were also included. Anemia was defined as hemoglobin < 130 g/L for men and hemoglobin < 120 g/L for women. N-terminal pro B-type natriuretic peptide and B-type natriuretic peptide plasma levels were dichotomized with cut-off values of 2000 pg/mL and 400 pg/mL, respectively. Using these cut-off points, three groups were established according to natriuretic peptide levels: low, high, and patients without data. Renal failure was defined as an estimated glomerular filtration rate < 60 mL/min/1.73 m^2^.

### 2.3. Follow Up

The vital status of patients at 6 months was verified by the researchers of each hospital. For this purpose, local health databases were used, or relatives were contacted.

### 2.4. Statistical Analysis

Continuous variables were expressed as the mean (95% confidence interval) or median (with 25th to 75th interquartile range), and categorical variables were expressed as frequencies and percentages. Continuous variables were compared using a Student’s *t*-test or non-parametric Kruskal–Wallis test. Categorical variables were compared using the Chi-square test. 

Both groups (acute HF with solid cancer versus without cancer) were compared. A multivariable logistic regression analysis was conducted, using the forward stepwise method. Variables tested included common prognostic markers, including the presence of a solid tumor cancer. A Kaplan–Meier survival analysis was performed to evaluate the impact of solid tumor cancer on the prognosis of patients with acute HF. Those with a statistical significance in the univariate analysis were included in the multivariate analysis. A *p*-value of less than 0.05 was considered statistically significant.

All analyses were performed with the Statistical Package for the Social Sciences (SPSS) program (version 26.0; SPSS, Armonk, NY, USA).

### 2.5. Ethical Aspects

The study was carried out in accordance with the Declaration of Helsinki. Ethical approval (Ethics Committee of the Hospital Virgen Macarena, Internal code 0942-N-15; 24 November 2015) was obtained before recruitment. All patients signed their informed consent at inclusion.

## 3. Results

A total of 3127 patients were included, of which 394 patients (13%) had a history of some type of cancer. The mean age of the latter was 79.29 +/− 10.2 years, with a predominance of the male sex (63%). The mean left ventricular ejection fraction was 53.28% +/− 15.78, and the mean NT-proBNP was 8933.62 pg/mL +/− 1047.51. Of the patients, 21.4% were in NYHA functional class III–IV. Their mortality during the follow-up period was 36%, which was 132 of the 162 patients who completed follow-up. (Table 1)

Patients with a history of cancer presented a greater frequency of weight loss at admission: 18% vs. 12% of patients without a history of cancer (*p* = 0.030). In the cancer group, functional impairment was more frequently observed (43% vs. 35%, *p* = 0.039). No significant statistical differences were observed between groups in relation to dyspnea, chest pain, nausea, insomnia, anguish, delirium, anxiety, and generalized pain. (Table 2) However, in patients with a history of cancer, the form of presentation of acute HF was 78/385 (20%) in the form of acute pulmonary edema vs. 408/2603 (16%) in patients without solid tumors (*p* = 0.026). Patients with a history of solid tumors more frequently presented with acute HF with preserved ejection fraction (65% vs. 58%, *p* = 0.048) than reduced or mildly reduced.

Table 3 shows the treatment received during admission in both groups of patients. Patients with a history of cancer were more frequently evaluated by the palliative care service (46% vs. 38%, *p* = 0.026), and in this group, the doses of oral morphine administered during admission were higher (32% vs. 26%, *p* = 0.42). No statistically significant differences were found between both groups in the administration of furosemide doses, use of amines, and non-invasive mechanical ventilation.

The in-hospital and six-month follow-up mortality was 31% (110/357) in patients with solid tumor vs. 26% (637/2466) without solid tumor (*p* = 0.046). 

**Figure 1:**
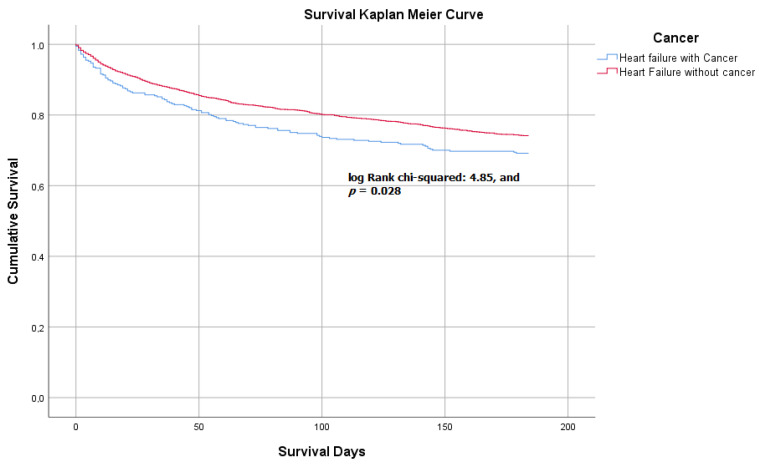
Kaplan–Meier survival curve between acute heart failure in patients with and without cancer.

The survival analysis showed that patients with a history of solid tumor (group 1) had fewer survival days during follow-up than those without solid tumor (group 2) (144.88 +/− 65.07 vs. 153 +/− 58.06 days, log Rank chi-squared: 4.85, and *p* = 0.028). (Figure 1)

## 4. Discussion

The results of our investigation show that in-hospital mortality and mortality during six-month follow-up in patients with acute HF admitted to internal medicine services were higher in those subjects with a history of cancer. A Danish study showed that all-cause mortality was higher in HF patients with cancer compared with cancer patients from the background population without HF: 1.24 (95% CI 1.15–1.33, *p* < 0.0001) (8). Hasin et al. [9] also reported a 60% increased risk of cancer in a series of American patients with HF. The study also reported that incident cancer was associated with a large excess risk of death (HR 1.68; 95% CI 1.33–2.14). Of note, this association was also observed after adjustment for age, sex, index year, and Charlson comorbidity index (HR 1.56; 95% CI 1.22–1.99) [9]. Compared to our study, patients were younger in both studies; mean age was 67.8 ± 2.2 and 75.5 ± 12.7 years, respectively [8,9], and they were not currently hospitalized with acute HF. 

We observed a male sex predominance in patients with acute HF and cancer, in contrast to the greater frequency of the female sex in the series studied on HF with preserved ejection fraction in internal medicine services in Spain [10]. This could be due to a higher prevalence of certain tumors such as lung, prostate, and genitourinary tumors in men. Men are more likely to develop cancer than women and have a poorer prognosis and an increased risk of secondary malignancies compared to women [10]. Thus, sex is associated with the prevalence of neoplasm and also contributes to the HF phenotype and incidence. Of note, men are generally diagnosed with HF at an earlier age, and they are also more frequently diagnosed with cancer [11]. In patients with cancer and acute heart failure, the mean left ventricular ejection fraction was > 50%, which may be explained by the fact that these patients share common risk factors such as advanced age, diabetes, or obesity [7].

In relation to the clinical manifestations, we found a higher frequency of weight loss and functional deterioration in patients with HF and cancer. Various factors have been associated with weight loss in HF patients, such as anorexia, poor nutrient absorption, shortness of breath, and increased inflammatory cytokines [12,13]. There is a generalized loss of adipose, osseous tissue, and muscle mass importantly affecting the skeletal muscle [14]. Hypoxia is a determining factor for the development of these metabolic alterations and is likely the main stimulus for increased TNF production in HF patients. Therefore, hypoxia leads to a hypercatabolic and inflammatory state [12]. This proinflammatory state also contributes to weight loss in patients with cancer, in which, as in HF, increased levels of tumor necrosis factor play a key role [15].

The findings of our investigation show that patients with acute HF and cancer were evaluated more frequently by the palliative care team in contrast to those without this condition. The history of cancer possibly influenced this assessment, despite the fact that many patients admitted for acute HF do so in an advanced clinical state. It seems likely that the management of cancer is less aggressive in patients with acute HF, as they are expected to tolerate chemotherapy less well than patients without HF.

Our results are in agreement with other authors, although HF is generally considered as a serious condition and equivalent to malignant disease in terms of symptom burden and mortality. Only a few patients receive specialist palliative care [16,17]. This could justify the higher use of morphine in patients with cancer and HF versus the other group. However, evidence indicates that a palliative approach in HF significantly improves patient outcomes, including symptom control and mental health, decreased hospital admissions and mortality, and reduced healthcare costs [18,19].

The present study has some limitations. The variable cancer was considered as an antecedent in the EPICTER study data collection. In this regard, we do not know the type of cancer at the time of inclusion, the stage of treatment, or whether the patient was in palliative care or in remission. The 5% mortality difference observed could have emerged from a particular solid cancer type or from a group of patients with no prior palliative care. These aspects are important when interpreting our results.

## 5. Conclusions

Patients with HF and cancer have worse survival than patients with HF and no cancer. However, the prognosis of patients admitted to our setting was poor, and many patients with HF may not receive palliative care assessment and support. This is a clinical care aspect to be improved and evaluated in future research studies.

## Figures and Tables

**Table 1:** Clinical characteristics and mortality of patients with acute heart failure with/without history of cancer.

Variable	Acute HF with Cancer (*n* = 394)	Acute HF without Cancer (*n* = 2733)	*p*
Age	79.29 +/− 10.2	78.83 +/− 11.05	*p* = 0.439
Sex (male)	247 (63%)	1295 (47%)	*p* < 0.001
NYHA III-IV	82/383 (21%)	639/2683 (24%)	*p* = 0.334
LVEF	53.28% +/− 15.78	50.63% +/− 16.14	*p* = 0.018
NTpro-BNP	8933 pg/mL +/− 1247	8334 pg/mL +/− 349	*p* = 0.561
Charlson score	4.42 +/− 1.7	3.44 +/− 1.86	*p* < 0.001
Comorbid conditions			
Hypertension	332/393 (85%)	2322/2726 (85%)	*p* = 0.705
Diabetes	168/392 (43%)	1257/2724 (46%)	*p* = 0.23
Ischemic cardiopathy	113/390 (29%)	885/2698 (33%)	*p* = 0.13
Atrial fibrillation	219/393 (56%)	1556/2723 (57%)	*p* = 0.624
Valvulopathy	176/377 (47%)	1141/2621 (44%)	*p* = 0.267
COPD	104/386 (27%)	704/2699 (26%)	*p* = 0.711
Chronic renal failure	210/393 (53%)	1254/2708 (46%)	*p* = 0.009
Previous cerebrovascular disease	75/392 (19%)	592/2700 (22%)	*p* = 0.237
Anaemia	211/394 (54%)	1302/2713 (48%)	*p* = 0.040
In-hospital and 6-month follow-up mortality	110/3573 (31%)	637/2466 (26%)	*p* = 0.046

Legend: NYHA: New York Heart Association functional class; LVEF: left ventricular ejection fraction; COPD: chronic obstructive pulmonary disease.

**Table 2:** Clinical characteristics of patients with and without cancer in patients with acute heart failure.

Variable	Acute HF with Solid Tumor	Acute HF without Solid Tumor	*p*
Weight loss	29/161 (18%)	143/1227 (12%)	*p* = 0.03
Functional decline	78/181 (43%)	461/1313 (35%)	*p* = 0.039
Dyspnea	208/261 (80%)	1375/1767 (78%)	*p* = 0.52
Anxiety	87/621 (33%)	572/1767 (32%)	*p* = 0.77
Nausea	36/261 (14%)	197/1767 (11%)	*p* = 0.021
Chest pain	49/261 (19%)	333/1764 (19%)	*p* = 1.00
Generalized pain	75/261 (29%)	478/1285 (27%)	*p* = 0.603
Delirium	40/261 (15%)	262/1767 (15%)	*p* = 0.85
Insomnia	97/261 (37%)	613/1765 (35%)	*p* = 0.445

**Table 3:** Treatment received during admission in both groups.

Variable	Acute HF with Solid Tumor (*n* = 394)	Acute HF without Solid Tumor (*n* = 2733)	*p*
Intravenous furosemide	98.5%	97.4%	*p* = 0.394
Noninvasive mechanical ventilation	5%	5%	*p* = 0.423
Hypertonic saline + furosemide	2%	3%	*p* = 0.394
Use of amines	5%	5%	*p* = 0.618
Vasodilators	9%	11%	*p* = 0.892
Dialysis	1%	1%	*p* = 0.987
Oral morphine	32%	26%	*p* = 0.045
Intravenous morphine	12%	11%	*p* = 0.823
Benzodiazepines	36%	33%	*p* = 0.405

## Data Availability

https://www.mdpi.com/ethics (accessed on 17 January 2022).
